# Oral Probiotics, *Streptococcus salivarius* K12 and M18, Suppress the Release of Volatile Sulfur Compounds and a Virulent Protease from Oral Bacteria: An In-Vitro Study

**DOI:** 10.3290/j.ohpd.b4328987

**Published:** 2023-08-29

**Authors:** Ji-A Park, Gyo Rin Lee, Jae-Young Lee, Bo-Hyoung Jin

**Affiliations:** a Senior Researcher, Department of Preventive Dentistry and Public Oral Health, School of Dentistry, Seoul National University, Seoul, Korea; Dental Research Institute, School of Dentistry, Seoul National University, Seoul, South Korea. Study concept and design, performed the experiments, interpreted the data, drafted and critically revised the manuscript.; b Master’s Student, Department of Preventive Dentistry and Public Oral Health, School of Dentistry, Seoul National University, Seoul, Korea; Dental Research Institute, School of Dentistry, Seoul National University, Seoul, South Korea. Study concept and design, performed the experiments, interpreted the data.; c Assistant Professor, Department of Dental Hygiene, College of Health Science, Dankook University, Cheonan-si, South Korea. Study concept and design, performed the experiments, i interpreted the data, drafted and critically revised the manuscript, gave final approval of the version to be published.; d Professor, Department of Preventive Dentistry and Public Oral Health, School of Dentistry, Seoul National University, Seoul, Korea; Dental Research Institute, School of Dentistry, Seoul National University, Seoul, South Korea. Study concept and design.

**Keywords:** gingipain, oral malodour, probiotics, *Streptococcus salivarius* K12, *Streptococcus salivarius* M18

## Abstract

**Purpose:**

To evaluate the inhibitory effects of *Streptococcus salivarius* K12 and M18 strains on the growth of six oral pathogens as well as their release of volatile sulfur compounds (VSCs), and whether these probiotics can inhibit the expression of arginine-specific gingipain A (RgpA), a protease secreted by *Porphyromonas gingivalis*.

**Materials and Methods:**

After six halitogenic oral pathogens (*P. gingivalis, Treponema denticola, Tannerella forsythia, Fusobacterium nucleatum, Parvimonas micra,* and *Eikenella corrodens*) were cultured with or without *S. salivarius* K12 and M18, the concentration of two VSCs was measured. Moreover, the antimicrobial activity of *S. salivarius* K12 and M18 against these pathogens and the suppressive effect on RgpA release by *P. gingivalis* were assessed.

**Results:**

In the co-culture of *S. salivarius* K12 or M18 with oral pathogenic bacteria, the growth of all six oral pathogens was significantly inhibited (p < 0.01). Additionally, *S. salivarius* K12 and M18 had an inhibitory effect on the production of the halitogenic substances H2S and CH3SH (p < 0.01) as well as the expression of *P. gingivalis* RgpA. Finally, we demonstrated that the addition of only culture supernatants of the two strains K12/M18 to oral pathogen cultures was sufficient to mimic the effects of K12/M18 co-cultures upon VSCs production and protease expression.

**Conclusions:**

*S. salivarius* K12 and M18 inhibited VSC release by all six of the major oral pathogens that were assayed and reduced the expression of RgpA.

The oral cavity is a favourable environment for microbial colonisation. Numerous bacteria live as dental biofilms on tooth surfaces in the oral cavity. They are also found on the mucosa and tongue, and in saliva. Periodontitis is affected by harmful substances produced by oral bacteria.^20^ As periodontal disease progresses, the pattern of bacterial colonisation of the gingival sulcus and tooth surface changes.^22^ Dental biofilms are involved in the pathogenesis of periodontal diseases and are composed of various bacteria, such as *Porphyromonas gingivalis, Treponema denticola, Tannerella forsythia*, and other gram-negative anaerobes, which generate lipopolysaccharide-producing tissue-destructive enzymes or proinflammatory cytokines.^21,22,27^

These periodontal disease-related bacteria are associated with halitosis, a health concern in modern populations. For example, the levels of *T. denticola* and *T. forsythia* were higher in the saliva of periodontitis patients with oral malodour than in non-periodontitis patients.^24^ These anaerobes use proteolytic enzymes in energy metabolism to produce volatile sulfur compounds (VSCs), which are the main cause of oral malodour. Even at low concentrations, VSCs adversely affect periodontal cells and facilitate bacterial invasion; thus, they may act as virulence factors in periodontal diseases.^11,19^

Probiotics are microorganisms or their products that have beneficial effects on health when consumed in appropriate amounts. Probiotics that can improve oral health, such as *Lactobacillus* or *Streptococcus* spp., have been previously studied.^13^

The gram-positive facultative anaerobe *S. salivarius* is an oral microbe that secretes a bacteriocin-like inhibitory substance (BLIS), a type of antimicrobial peptide that inhibits the growth of other bacteria.^29^ Among *S. salivarius* strains, *S. salivarius* K12 and M18 exhibit broad-spectrum bacteriocin effects.^6,8^ They can resist pneumococcal species (M18), inhibit the adhesion of harmful bacteria to the oropharyngeal epithelium (K12), prevent throat infection and tonsillitis (K12),^3,14,25^ and inhibit the growth of *Candida* (K12) and caries-causing bacteria (M18) in the oral cavity.^4,7^ In other studies, *S. salivarius* K12 and M18 decreased interleukin (IL)-6 and IL-8 secretion in gingival fibroblasts challenged by *P. gingivalis, Aggregatibacter actinomycetemcomitans,* and *Fusobacterium nucleatum,*^13^ and inhibited the toxicity of these bacteria in oral keratinocytes.^17^ Some probiotics, including *S. salivarius* K12 and M18, have exhibited synergistic activity in suppressing the growth of periodontal bacteria.^10^ Thus, *S. salivarius* K12 and M18 have the potential to prevent and suppress periodontal diseases.

Several studies have confirmed that these microbes affect the components of oral malodour. *S. salivarius* K12 inhibited the production of VSCs by gram-positive halitosis-causing bacteria in clinical trials with small sample sizes and in in-vitro studies.^2,15^ In other studies, *S. salivarius* K12 and M18 inhibited the growth of halitogenic gram-negative anaerobes, such as *Prevotella intermedia, P. gingivalis*, and *T. denticola*, and the secretion of halitosis-related materials.^18,28^ In another clinical trial, VSC levels were reduced and the bacterial composition changed when participants with orthodontic appliances ingested *S. salivarius* M18 lozenges,^1^ and *S. salivarius* K12 reduced oral malodour in the organoleptic test.^9^

Several oral bacteria are periodontopathic and halitogenic. However, many studies on the effect of probiotics focused either on periodontal diseases or on halitosis, not both. To explore the therapeutic potential of the two probiotics, it is imperative to evaluate their capacity to simultaneously suppress both periodontal disease and oral malodour. Therefore, this study aimed to investigate the simultaneous effects of *S. salivarius* K12 and M18 on periodontal disease and halitosis. Thus, we comprehensively evaluated the inhibitory effects of *S. salivarius* K12 and M18 on the growth of six oral pathogens as well as the impact on their release of VSCs. These pathogens – including periodontal bacteria such as *P. gingivalis, T. denticola, T. forsythia,* and *F. nucleatum*, and other harmful bacteria such as *Parvimonas micra* and *Eikenella corrodens* – are mainly related to periodontal disease and produce VSCs. In this study, a modified method was used to measure the concentration of VSCs in vitro. In addition, this study confirmed the effects of two probiotics on the major periodontal pathogen *P. gingivalis* by comprehensively investigating their effects on the various virulence factors of *P. gingivalis* related to periodontal damage and halitosis. Thus, we also examined whether these probiotics could inhibit the expression of arginine-specific gingipain A (RgpA), a representative proteolytic enzyme of *P. gingivalis* that degrades periodontal tissue and releases VSC.

## Materials and Methods

This study did not use human samples; therefore, ethical approval and consent to participate were not required.

### Bacterial Cultures and Supernatant Preparations

We used two probiotic strains, *S. salivarius* K12 (Juyeongns; Seoul, South Korea) and *S. salivarius* M18 (Juyeongns). Further, the following six oral pathogenic bacteria were selected because of their relationship to periodontal diseases and halitogenic substances: *P. gingivalis* from the Korean Collection for Type Cultures (KCTC) 5352 (KCTC, Korea Research Institute of Bioscience and Biotechnology; Jeongeup, Korea); *T. forsythia* (KCTC 5666); *T. denticola* (KCTC 15104) from the red complex, which is most closely related to periodontitis; *F. nucleatum* (KCTC 2640) from the orange complex; *P. micra* (KCTC 15021); and *E. corrodens* KCTC 15198 from the green complex.^22^ *S. salivarius* K12 and M18 were cultured in brain-heart infusion (BHI) medium. *P. gingivalis, F. nucleatum*, and *P. micra* were cultured in BHI medium containing hemin and vitamin K3. *E. corrodens* was cultured in BHI medium containing fetal bovine serum. In an anaerobic environment, *T. forsythia* was cultured in a new oral spirochete (NOS) medium and *T. denticola* was cultured in NOS medium containing vitamin K3 and N-acetylmuramic acid.

To confirm the effect of the probiotics *S. salivarius* K12 and M18 and their metabolites, cultured supernatants of the two strains were isolated. The cells were filtered through a 0.20-µm pore-size filter (SC25PO20SS, Hyundai Micro; Seoul, South Korea) from medium containing 4 x 10^8^ colony-forming units (CFUs) of *S. salivarius* obtained after culturing for 24 h, and only the supernatant was extracted. It was diluted to half strength in BHI medium. The strain itself was used for experiments involving six oral pathogenic bacteria. The conditions for each bacterial experiment were set by using the optimal number of passages and quantifying the number of bacteria per time period through optical density measurement to provide basic data.

### Inhibition of Bacterial Growth

Antimicrobial susceptibility testing was performed following the Clinical and Laboratory Standards Institute (CLSI) protocols.^28^ Each probiotic strain, *S. salivarius* K12 or M18, at a concentration of 1 x 10^8^ CFUs, was mixed with six types of oral pathogenic bacteria at 1:1, 1:5, and 1:10 concentrations and incubated for 36 h on Millicell hanging inserts (Merck; Darmstadt, Germany). To evaluate whether colonies were formed during co-culture and whether contamination occurred, the degree of colony formation was determined using phase-contrast microscopy.

We prepared *S. salivarius* K12 and M18 supernatants, diluted them with equal amounts of the six oral pathogens in a 96-well plate using the double serial dilution method recommended by the Clinical and Laboratory Standards Institute, and incubated them for 36 h after supernatant injection.

After co-culturing a probiotic strain (either K12 or M18) or its supernatant with six oral pathogens, we quantified bacterial growth by measuring optical absorbance at 600 nm and compared this with standard growth curves for each bacterium to evaluate the degree to which bacterial growth was affected. All experiments were repeated in triplicate, and average values of the three experiments were calculated.

### Suppression of VSC Production by Bacteria

To evaluate the concentration of VSCs in the culture solution, a special sterilised glass was used for gas measurements. In the experiment involving the co-culture of probiotics and oral pathogens, experimental groups were prepared by adding either *S. salivarius* K12 or M18 to glass chambers containing each oral pathogen (oral pathogens to probiotic ratios assayed were 1:1, 1:2, and 1:3) and mixed for 30 s using a vortex mixer (Vortex-GENIE 2, Scientific Industries; Bohemia, NY, USA). The resultant mixture was sealed and co-cultured for 30 min in an anaerobic chamber. The control groups consisted of BHI-only medium and single oral bacterial culture solutions. The total volume of culture solution in each glass was 10 ml.

In the experiment using *S. salivarius* K12 and M18 supernatants, the experimental group was prepared by configuring the volume ratio between the oral bacterial culture medium and the probiotic supernatant to 1:1, 1:2, and 1:3. Cells were further cultured for 30 min in the same manner as that used for the bacterial co-culture. The total volume of culture solution in each glass was 5 ml.

We collected oral bacteria-produced VSCs and determined their concentrations using a method modified from previous studies.^18,28^ We measured two VSC species, hydrogen sulfide (H_2_S) and methyl mercaptan (CH_3_SH). Gases (10 ml) were drawn from a sealed glass chamber with a gas-tight syringe to avoid leakage, and diluted 10 times with normal air. VSC concentrations were measured using a Twin Breasor II (iSenLab; Seoul, South Korea). All experiments were repeated in triplicate, and average value was calculated.

### Inhibition of the Release of Bacterial Protease

To evaluate whether *S. salivarius* K12 and M18 could inhibit the expression of RgpA (a virulence factor of *P. gingivalis* that disrupts host tissue invasion and defense mechanisms), *S. salivarius* K12 and M18 were co-cultured with *P. gingivalis* at the same concentration under anaerobic conditions for 24 h. A co-culture group of probiotics and *P. gingivalis* was used as the experimental group and a group comprising only *P. gingivalis* culture was used as the control group. After the mRNA secreted by the bacteria was extracted from cultures, complementary DNA was synthesised using reverse transcriptase; the mRNA component of *rgpA* gene was detected using real-time polymerase chain reaction (PCR). Real-time PCR was performed for 40 cycles of denaturation at 95°C, annealing at 60°C, and extension at 72°C.

### Statistical Analysis

Statistical analyses were performed using SPSS 25.0 (IBM; Armonk, NY, USA), and GraphPad Prism version 5.01 software (GraphPad Software; San Diego, CA, USA, RRID:SCR_002798). The degree of growth inhibition of the six oral pathogens was determined by comparing the absorbances of the six cultured individually with those co-cultured with *S. salivarius* K12/M18 or with K12/M18 supernatants. VSCs inhibition was compared with the concentrations of each gas (H_2_S and CH3SH) measured using a specialised device. Differences between control and experimental groups were analysed using one-way ANOVA and Tukey’s post-hoc test (statistical significance set at p < 0.001). The inhibitory effect on *rgpA* gene expression in *P. gingivalis* culture was quantified by amplifying secreted mRNA using real-time PCR. Real-time PCR data analyses were performed using ABI PRISM 7500 software v2.06 (Applied Biosystems; Foster City, CA, USA).

## Results

The datasets generated from the present study are available from the corresponding author upon reasonable request.

### *S. salivarius* K12 and M18 Inhibited the Growth of Oral Pathogenic Bacteria

**Fig 1:**
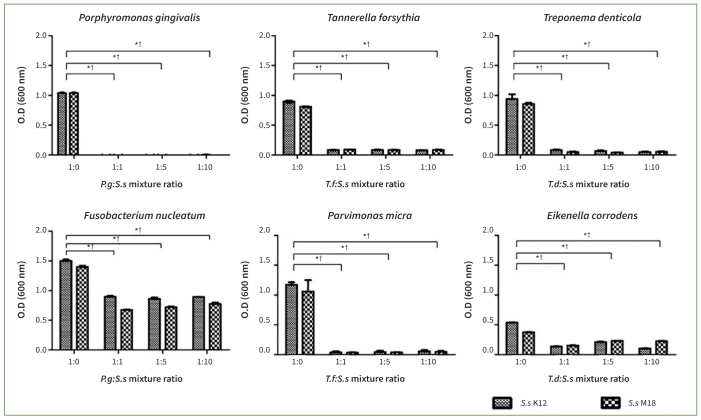
*Streptococcus salivarius* (*Ss*) K12 and M18 inhibited the growth of oral pathogenic bacteria in co-culture. In all pathogenic bacteria co-cultured with *Ss* K12 or M18 (*Porphyromonas gingivalis* [*Pg*], *Tannerella forsythia* [*Tf*], *Treponema denticola* [*Td*], *Fusobacterium nucleatum* [*Fn*], *Parvimonas micra* [*Pm*], and *Eikenella corrodens* [*Ec*]), their growth was statistically significantly suppressed at all concentrations assayed. Bars and error bars present means ± SD of optical density readings, which reflect the viability of each oral pathogenic bacterial strain. *p< 0.001 and †p< 0.001: statistically significantly different from the control (a 1:0 ratio) in co-cultures of *Ss* K12 and M18, respectively. Each point was measured in triplicate.

The antimicrobial efficacy results for *S. salivarius* K12 and M18 are presented in Fig 1. In the co-culture of *S. salivarius* K12 or M18 with oral pathogenic bacteria, the growth of all species tested in the experiment, including *P. gingivalis, F. nucleatum, E. corrodens, T. denticola, T. forsythia,* and *P. micra*, was statistically significantly suppressed at all concentrations (p < 0.001). The degree of colony formation on the plate was observed under a microscope to indirectly examine the effect of probiotics on bacterial colonisation in the oral cavity. We found that colony formation was statistically significantly reduced when probiotics and oral bacteria were mixed, compared to individually culturing the 6 oral bacteria species (not shown in Fig 1).

**Fig 2:**
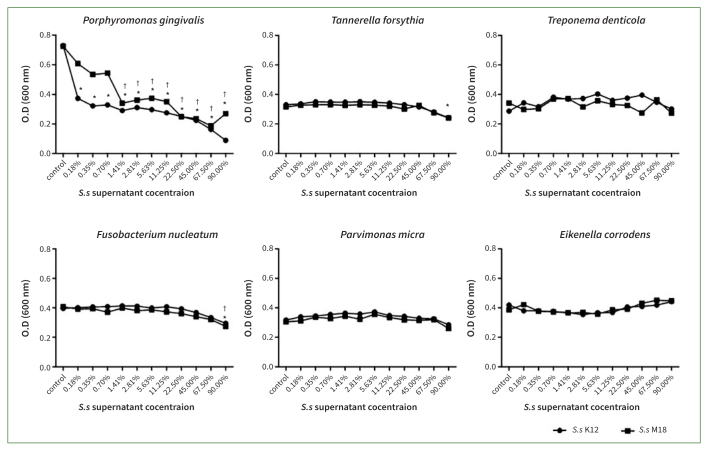
The addition of *Streptococcus salivarius* (*Ss*) K12 and M18 supernatants to oral pathogenic bacteria cultures statistically significantly inhibited the growth of several pathogenic bacteria assayed. Although the growth of *Porphyromonas gingivalis* (*Pg*) was significantly inhibited by very low concentrations of *Ss* K12 or M18 supernatants, the growth of *Tannerella forsythia* (*Tf*) and *Fusobacterium nucleatum* (*Fn*) was statistically significantly reduced only when supernatant was provided at its highest concentration (90% [v/v]). Decreases of *Treponema denticola* (*Td*), *Parvimonas micra* (*Pm*), and *Eikenella corrodens* (*Ec*) were not statistically significant. *p< 0.001 and †p< 0.001 indicate a statistically significant difference from the control after addition of *Ss* K12 and M18 supernatants, respectively.

The addition of *S. salivarius* K12 and M18 supernatants inhibited the growth of some oral pathogenic bacteria (Fig 2). The growth of *P. gingivalis* was statistically significantly inhibited by the lowest concentration of the supernatant of the two species (p < 0.001), wherease the growth of *F. nucleatum* and *T. forsythia* was statistically significantly reduced when the supernatant was at the highest concentration (90% [v/v]). The decrease in the numbers of other oral bacteria was not statistically significant. The formation of colonies on the plate was substantially reduced even when the probiotic supernatant was applied (not shown in Fig 2).

### Co-culture with *S. salivarius* K12 or M18 Suppressed VSCs Production by Oral Pathogenic Bacteria

**Fig 3:**
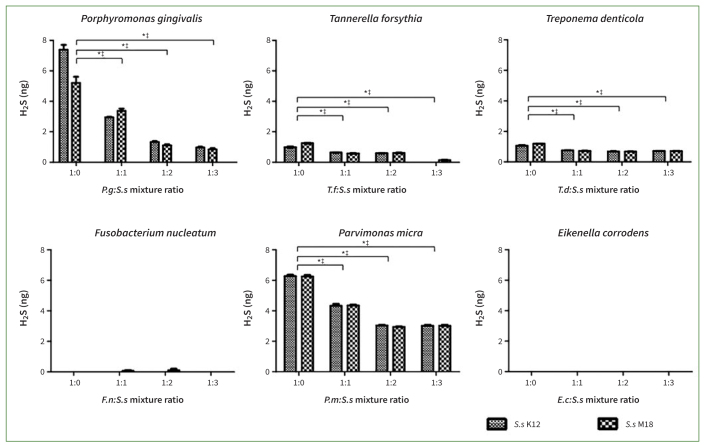
Co-culturing *Streptococcus salivarius* (*Ss*) K12 or M18 with oral pathogenic bacteria suppressed the production of hydrogen sulfide (H_2_S) gas. H_2_S gas was not produced by *Fusobacterium nucleatum* (*Fn*) and *Eikenella corrodens* (*Ec*), but was by the other oral bacteria assayed. Co-culturing with *Ss* K12 or M18 statistically significantly reduced concentrations of H_2_S gas released by *Porphyromonas gingivalis* (*Pg*), *Tannerella forsythia* (*Tf*), *Treponema denticola* (*Td*), and *Parvimonas micra* (*Pm*) in all ratios assayed (1:1, 1:2, and 1:3 (*Ss* K12 or M18: oral pathogenic bacteria), respectively. Bars and error bars present the mean ± SD of H_2_S (ng) gas concentration. *p< 0.001 indicate a statistically significant difference from the control (a ratio of 1:0) in co-cultures with *Ss* K12. †p< 0.01 and ‡p< 0.001 indicate a statistically significant different from the control (a ratio of 1:0) in co-cultures with *Ss* M18. Each point was measured in triplicate.

As shown in Fig 3, H_2_S gas was not produced in the experiments with *F. nucleatum* and *E. corrodens,* but was produced in the experiments with other oral bacteria. The H_2_S concentration was reduced by co-culturing with *S. salivarius* K12 or M18. When *S. salivarius* K12 or M18 was mixed with oral pathogenic bacteria, including *P. gingivalis, T. denticola, T. forsythia,* and *P. micra*, at concentrations of 1:1, 1:2, and 1:3, the H_2_S concentration decreased statistically significantly at all ratios (p < 0.01). When the ratio of probiotics to oral pathogens tripled, *S. salivarius* K12 almost completely blocked the generation of H_2_S in the *T. forsythia* group and reduced the H_2_S concentration by 87% in the *P. gingivalis* group. *S. salivarius* M18 maximally reduced H_2_S concentrations by up to 89%, 87%, and 51% in the *T. forsythia, P. gingivalis,* and *P. micra* groups, respectively.

**Fig 4:**
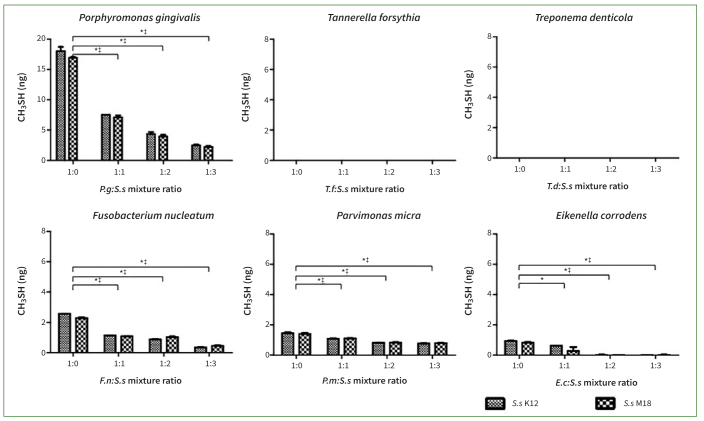
The co-culture of *Streptococcus salivarius* (*Ss*) K12 or M18 suppressed production of methyl mercaptan (CH_3_SH) gas by oral pathogenic bacteria. CH_3_SH gas was not produced in experiments with *Tannerella forsythia* (*Tf*) and *Treponema denticola* (*Td*), but was produced by the other oral bacteria assayed. Co-cultures with *Ss* K12 or M18 statistically significantly reduced the concentration of CH_3_SH gas released by *Porphyromonas gingivalis* (*Pg*), *Fusobacterium nucleatum* (*Fn*), *Parvimonas micra* (*Pm*) and *Eikenella corrodens* (*Ec*) in nearly every mixture ratio assayed (*Ss* K12 or M18:oral pathogenic bacteria at 1:1, 1:2, and 1:3, respectively). Bars and error bars present means ± SD of CH_3_SH (ng) gas concentrations. *p< 0.001: statistically significantly different from the control (the ratio of 1:0) in the co-culture of *Ss* K12. †p< 0.05 and ‡p< 0.001: statistically significantly different from the control (the ratio of 1:0) in co-culture of *Ss* M18. Each point was measured in triplicate.

CH_3_SH was not produced in the *T. denticola* and *T. forsythia* groups, but was released by the other four bacterial groups. The concentration of CH_3_SH was reduced in the *P. gingivalis, F. nucleatum, E. corrodens,* and *P. micra* groups in co-culture with *S. salivarius* K12 or M18 (p < 0.05). Notably, a reduction in the concentration of malodourous substances was observed in the *P. gingivalis, F. nucleatum,* and *E. corrodens* groups. When the ratio of probiotics to oral pathogens was tripled, *S. salivarius* K12 or M18 decreased the concentration of CH_3_SH by 96–98% in the *E. corrodens* group, 81–87% in the *P. gingivalis* and *F. nucleatum* groups, and 44%–46% in the *P. micra* group (Fig 4). These results confirm the inhibitory effect of *S. salivarius* K12 and M18 on the production of halitogenic substances.

### Cultured Supernatant of *S. salivarius* K12 or M18 Partially Suppressed the Production of VSCs by Oral Pathogenic Bacteria

**Fig 5:**
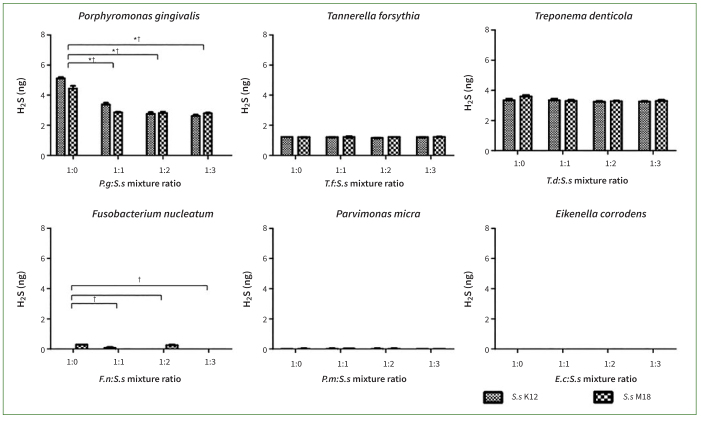
Culture supernatants of *Streptococcus salivarius* (*Ss*) K12 or M18 partially suppressed the production of hydrogen sulfide (H_2_S) gas by oral pathogenic bacteria. H_2_S gas was produced at a very low level in experiments with *Fusobacterium nucleatum* (*Fn*), *Parvimonas micra* (*Pm*), and *Eikenella corrodens* (*Ec*), but was robustly produced by *Porphyromonas gingivalis* (*Pg*), *Tannerella forsythia* (*Tf*), and *Treponema denticola* (*Tg*). Culture supernatants of *Ss* K12 or M18 statistically significantly reduced the concentration of H_2_S gas released by *Porphyromonas gingivalis* (*Pg*) only, in the ratios of 1:1, 1:2, and 1:3 (*Ss* K12 or M18:*Pg*). Bars and error bars present means ± SD of H_2_S (ng) gas concentrations. * p< 0.001 and †p< 0.001: statistically significantly different from the control (the ratio of 1:0) in the addition of supernatant of *Ss* K12 and M18, each. Each point was measured in triplicate.

**Fig 6:**
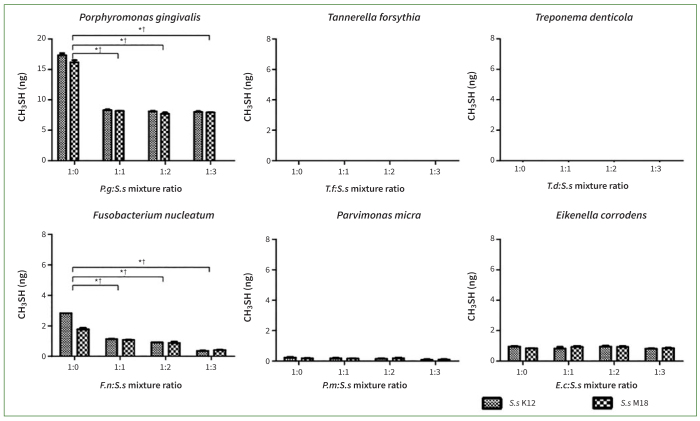
Culture supernatants of *Streptococcus salivarius* (*Ss*) K12 or M18 partially suppressed the production of methyl mercaptan (CH_3_SH) gas by oral pathogenic bacteria. CH_3_SH gas was not produced in experiments with *Tannerella forsythia* (*Tf*) and *Treponema denticola* (*Tg*) but was produced in experiments with other oral bacteria. As the concentration of K12 or M18 culture supernatant was increased, concentrations of CH_3_SH concomitantly decreased in *Porphyromonas gingivalis* (*Pg*) and *Fusobacterium nucleatum* (*Fn*) in all ratios assayed (1:1, 1:2, and 1:3 of Ss K12/M18:oral pathogenic bacteria, respectively). However, *Parvimonas micra* (*Pm*) and *Eikenella corrodens* (*Ec*) VSCs were not statistically significantly affected. Bar and error bar present the means ± SD of gas concentrations of CH_3_SH (ng). * p< 0.001 and †p< 0.001 indicate statistically significant differences from the control (a ratio of 1:0) in the addition of *Ss* K12 and M18 supernatants, respectively. Each point was measured in triplicate.

When the cultured supernatant of *S. salivarius* K12 or M18 was added to the oral pathogenic bacterial culture, the tendency was similar to that of co-culture with live strains in terms of the occurrence of each VSC and inhibition of VSCs. However, the decline in the concentration of VSCs in the supernatant setting was less than that in the co-culture setting (Figs 5 and 6). At baseline, H_2_S gas was barely detectable in the *F. nucleatum, E. corrodens,* and *P. micra* groups. The generation of H_2_S was blocked by up to 37–49% only in the *P. gingivalis, T. forsythia, and T. denticola* groups compared to that in the control group (p < 0.001). Only these 3 species produced substantial amounts of H_2_S, while the other species (*F. nucleatum, P. micra,* and *E. corrodens*) produced little or none.

As shown in Fig 6, CH_3_SH gas was not produced in the experiments with *T. denticola* and *T. forsythia* at baseline, but was produced in the experiments with the other four oral bacteria. As the concentration of the culture supernatant of *S. salivarius* K12 or M18 increased, the concentration of CH_3_SH gradually decreased in the four species, ie, the inhibitory effect on the generation of halitogenic substances increased. The generation of CH_3_SH was inhibited by up to 51–54% and 77–88% in the *P. gingivalis* and *F. nucleatum* groups, respectively (p < 0.01).

### *S. salivarius* K12 and M18 Inhibited the Expression of RgpA Induced by *P. gingivalis*

**Fig 7:**
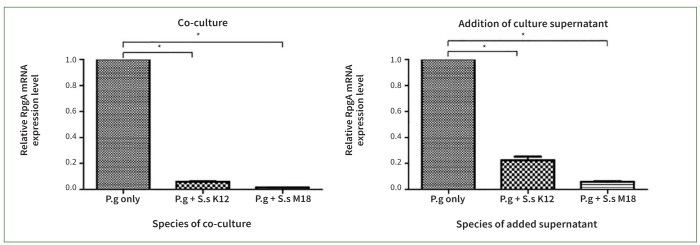
*Streptococcus salivarius* (*Ss*) K12 or M18 inhibited the expression of RgpA mRNA induced by *Porphyromonas gingivalis* (*Pg*). The amplification of RgpA mRNA released by *Pg* was compared among groups by real-time PCR system. The relative expression of RgpA mRNA was statistically significantly lower in co-cultures of *Pg* with *Ss* K12 or M18 than in the control group. Even when *P. gingivalis* was mixed with *Ss* K12 or M18 culture supernatants, the expression of RgpA mRNA was decreased. The suppression of RgpA mRNA by *Ss* M18 was greater than that by *Ss* K12. Bars and error bars present means ± SEM of relative RgpA mRNA expression levels. * p< 0.001 indicates a statistically significant difference from the control (*Pg* only). Each point was measured in triplicate.

The expression of RgpA of *P. gingivalis* was confirmed by extracting mRNA from cultures. As shown in Fig 7, the amplification of RgpA mRNA was compared with that expressed by individually cultured *P. gingivalis* as a control. The relative expression of RgpA mRNA was significantly lower in the co-culture of *P. gingivalis* with either *S. salivarius* K12 or M18 than in the control. Even when *P. gingivalis* was mixed with *S. salivarius* K12 or M18 culture supernatant, the expression of RgpA mRNA decreased (p < 0.001). Thus, *S. salivarius* K12 and M18 were both found to inhibit expression of *P. gingivalis*-derived RgpA. Interestingly, *S. salivarius* M18 had a greater inhibitory effect than did *S. salivarius* K12.

## Discussion

This study expanded the results of previous studies on *S. salivarius* K12 and M18, and evaluated the simultaneous effects of the two probiotics on periodontal disease and halitosis by targeting six oral pathogenic bacteria related to these conditions. As periodontal pathogens inevitably generate VSCs in the process of energy metabolism, their inhibition by oral probiotics has the advantage of simultaneously targeting periodontal disease and halitogenic components. Additionally, because VSCs can damage periodontal tissue, the study of bacteria inhibiting oral halitosis can have a positive effect on controlling periodontal disease.

Socransky et al^22,23^ classified subgingival bacterial colonies according to the development and severity of periodontitis as follows: red complex, comprising *P. gingivalis, T. denticola,* and *T. forsythia*; orange complex, comprising *F. nucleatum* and *P. micra*, which are highly important in periodontal disease; green complex, comprising *E. corrodens*, which is associated with periodontal disease. Most previous studies have mainly examined the effects of probiotics on the major periodontal bacteria *P. gingivalis, F. nucleatum*, and *Actinomyces* spp.^13,18,28^ However, we comprehensively reviewed previous studies on *S. salivarius* and also included different bacteria – *P. micra* and *E. corrodens* – as tested pathogens, while also expanding the scope to include the effect of probiotics on both periodontopathic and halitogenic characteristics. Thus, we evaluated the potential of *S. salivarius* M18 and K12 as simultaneous inhibitors of periodontal disease and halitosis.

By applying two oral probiotics, *S. salivarius* K12 and M18 or their culture supernatants to oral pathogens, it was confirmed that the probiotics inhibited the growth of all six oral pathogenic bacteria. The effect was more distinct when live probiotics and oral pathogens were co-cultured at varying concentrations (Fig 1). However, when applying the probiotics supernatants, statistically significant suppression of growth was observed only for some oral bacteria, such as *P. gingivalis*, and at very high concentrations of the supernatant (Fig 2).

Several substances secreted from cultured microorganisms are in cell-free supernatants, the identities of which are determined by the specific biological activities of their constituent bacteria.5 *S. salivarius* K12 and M18 secrete BLIS, which can inhibit the growth of other bacteria.^6,8,29^ Therefore, some studies have examined whether, in addition to live cells, culture supernatants of *S. salivarius* K12 and M18 can similarly exert inhibitory effects on other bacteria and mitigate inflammation caused by pathogen infections,^5,14^ or whether they can reduce halitogenic substances.^28^ The effects of probiotic supernatant have been inconsistent across studies and target bacteria. Unprocessed probiotic supernatant alone cannot inhibit pneumococcal growth;^14^ however, in a previous study, supernatant first fractionated into smaller pieces was able to reduce the expression of inflammatory cytokines.^13^ This study examined the effect of probiotic supernatants with reference to the methodology of previously mentioned studies and confirmed that probiotic supernatants also had inhibitory effects on bacterial growth, VSC levels, and enzyme concentrations. Especially the supernatant clearly inhibited the growth of *P. ginigivalis* and its VSC release. BLIS or similar substances present in the supernatant may have exerted an influence. However, the overall effect of supernatant inoculation was not as strong as that of live strain co-culture, similar to the results of other studies.^13,17^ Therefore, it can be inferred that the additional effect was due to direct interactions among the microorganisms.

In the present study, after we found that *S. salivarius* K12 and M18 strains or their supernatants could inhibit the growth of periodontal disease-related oral bacteria themselves, we next investigated whether the production of VSCs by these bacteria could also be inhibited. According to our results, the two probiotics reduced the production of substances that cause halitosis, including H_2_S and CH_3_SH. Although some species did not produce H_2_S or CH_3_SH per se, both the *S. salivarius* K12 and M18 strains were effective in reducing the concentration of halitogenic substances produced by bacteria; the co-culture with live probiotics was more effective than the addition of the supernatant. *S. salivarius* K12 and M18 showed distinct effects on the inhibition of H_2_S in the *T. forsythia* and *P. gingivalis* groups, as well as on the inhibition of CH_3_SH in the *E. corrodens, P. gingivalis,* and *F. nucleatum* groups (Figs 3 and 4). In previous studies, these probiotics reduced the generation of VSCs by gram-positive halitogenic bacteria, including *P. intermedia, P. gingivalis,* and *T. denticola*.^2,15,18,28^ By confirming that *S. salivarius* K12 and M18 effectively suppressed the growth of *T. forsythia, E. corrodens*, and *P. micra* as well as the release of VSCs, the present study inferred their broad influence. The results obtained by adding the probiotic supernatant were consistent with those observed by co-culturing with live strains; however, the inhibitory effect of supernatants on VSCs as well as bacterial growth was not statistically significant, except for *P. gingivalis* and *F. nucleatum*.

As mentioned above, both live probiotics and supernatant were most effective against *P. gingivalis*, a representative bacterium that induces periodontal disease and produces VSCs. Considering that the growth of *P. gingivalis* itself was statistically significantly inhibited by *S. salivarius* K12 and M18 compared to that of other oral pathogenic bacteria, it can be inferred that live probiotics or BLIS secreted by them are particularly effective in controlling the growth of *P. gingivalis*, suppressing the release of VSCs and energy metabolites by this bacterium. These results correspond with those of other studies that have confirmed the effect of probiotics own *P. gingivalis*.^10,28^ Moreover, this study goes further to show that the supernatant of probiotics can also inhibit release of VSCs by *P. gingivalis*. Other studies examined the inhibitory effect of probiotics on the release of VSCs only in a co-culture set-up.

Expanding on the findings of previous studies on the interaction of *S. salivarius* K12 and M18 with *P. gingivalis*, the present study also examined their inhibitory effects on proteases, which are key virulence factors of periodontal pathogens. One study reported that these probiotics reduced the expression of pro-inflammatory cytokines by bacteria.^13^ However, the literature contains no study which examined the effect of *S. salivarius* K12 or M18 on proteases secreted by periodontal pathogens. We investigated whether these probiotics could affect the expression of RgpA (arginine-specific gingipain A), a representative proteolytic enzyme of *P. gingivalis* that plays an important role in the infiltration and disintegration of periodontal tissue. The expression of RgpA mRNA was reduced by *S. salivarius* K12 and M18 (Fig 7). Thus, it was confirmed that these two probiotics have the potential to inhibit the production of halitogenic and periodontal tissue-disintegrating substances by oral pathogens. The expression of secreted enzymes is also partially suppressed, as the growth of *P. gingivalis* is suppressed by these probiotics. However, to the best of our knowledge, this is the first study to confirm the inhibitory effects of *S. salivarius* K12 and M18 on the secretion of gingipain by *P. gingivalis*.

To recapitulate, *S. salivarius* K12 and M18 inhibited the growth of *P. gingivalis*, reduced the concentration of VSCs (which are toxic to periodontal tissues and cause halitosis), and suppressed the expression of a strong virulence factor of this bacterium. This suggests that the application of *S. salivarius* K12 and M18 in clinical practice can simultaneously target periodontal disease and halitosis, which are major problems in adult dental patients, and could even be used to prevent periodontal disease and oral malodour. In particular, it might be more effective in subjects with the high level of oral *P. gingivalis*.

### Strengths of the Study

Our study confirms the broad applicability of *S. salivarius* K12 and M18 probiotic strains as well as their culture supernatants in reducing numerous oral pathogens. This has not been characterised in any previous studies.

In the case of *P. gingivalis*, K12 and M18 distinctly inhibited the growth and release of VSCs. Additionally, the two probiotics also suppressed the secretion of gingipain by *P. gingivalis*. Gingipain is a strong virulence factor involved in bacterial adhesion, periodontal tissue inflammation and disintegration. The supernatant condition of probiotics was also effective against *P. gingivalis*. Overall, our findings lend support to the possibility that *P. gingivalis* could be significantly weakened by different forms of probiotics.

The present study clearly shows the potential of *S. salivarius* K12 and M18 to simultaneously target multiple major oral problems, including periodontal disease and halitosis.

### Limitations of the Study

This study had several limitations. First, because of the characteristics of microbial culture, it was not possible to standardise bacterial growth and the concentrations of excreted substances under the same conditions. The release of VSC by *P. micra* in particular was inconsistent in tests. This may have contributed to differences between our results and those of previous studies.

Second, the probiotic strains and their processed forms, such as an extract, can be used in clinical applications. However, in the present study, we examined the effects of the processed form of probiotic supernatants on the inhibition of oral pathogenic bacteria and found the effect size to be relatively weak or insignificant. To generate a biologically relevant effect size from treatment with substances such as BLIS secreted from probiotics, supernatant collection conditions and post-treatment conditions may need to be adjusted. Additionally, it is possible that the inhibitory effects we observed were stronger than expected because bacteria can interact directly in in-vitro co-culture conditions.

Meanwhile, our measurements were restricted to only two VSCs. In reality, in-vivo oral malodour is attributable to numerous additional substances and factors. Currently, there is a limited assortment of devices facilitating the measurement of halitogenic components; however, in the future, we expect the development of high-throughput measurement tools for such substances to revolutionise and deepen studies of factors produced by oral pathogens.

One other major drawback to our findings is that this study could not test the effect of probiotics on adhesion, aggregation, biofilm formation or its virulence in the mixed condition with several pathogens. Most oral pathogens interact and are more virulent as a biofilm. If the probiotics can block the process of adhesion or biofilm formation, they will be able to fundamentally prevent the occurrence of periodontal inflammation or malodour caused by these pathogens. Therefore, it is also important to evaluate the effect of probiotics on biofilm, rather than just individual pathogens. However, we only tested one bacterial species at a time. It was difficult to grow the tested periodontal bacteria together in an experimental environment, as they were absolute anaerobes. Most of the studies on the inhibition of biofilm formation by *S. salivarius* K12 and M18 have been on *C. albicans* or cariogenic bacteria such as *S. mutans*. However, they revealed that candidiasis or caries can be inhibited due to the probiotics influencing aggregation-related genes such as *gtf* genes.^12,16^ Furthermore, a recent report cultured periodontal pathogens, commensal bacteria, and probiotics together to form a periodontopathic biofilm, and evaluated the ability of probiotics to inhibit periodontal pathogens.^26^ The results for RgpA were also considered in our study, as RgpA is also involved in adhesion or aggregation of *P. gingivalis*. With this background, further studies are needed to co-culture several microbes and evaluate the effect of these probiotics on biofilm formation or VSCs production and other virulence expression in the biofilm state.

## Conclusion

*S. salivarius* K12 and M18 strains and their supernatants not only inhibited the growth of major oral pathogenic bacteria related to both periodontal disease and halitosis, but also reduced the generation of VSCs, which are representative oral malodour-inducing substances. Furthermore, they reduced the expression of proteolytic enzymes in the major periodontal pathogen, *P. gingivalis*. Thus, the potential of *S. salivarius* K12 and M18 to target major oral problems, such as periodontal disease and halitosis, was confirmed.
